# An overview of computational methods in single-cell transcriptomic cell type annotation

**DOI:** 10.1093/bib/bbaf207

**Published:** 2025-05-10

**Authors:** Tianhao Li, Zixuan Wang, Yuhang Liu, Sihan He, Quan Zou, Yongqing Zhang

**Affiliations:** School of Computer Science, Chengdu University of Information Technology, No. 24 Block 1, Xuefu Road, 610225 Chengdu, China; College of Electronics and Information Engineering, Sichuan University, No. 24 South Section 1, 1st Ring Road, 610065 Chengdu, China; Faculty of Applied Sciences, Macao Polytechnic University, 999078 Macao, China; School of Computer Science, Chengdu University of Information Technology, No. 24 Block 1, Xuefu Road, 610225 Chengdu, China; Institute of Fundamental and Frontier Sciences, University of Electronic Science and Technology of China, Shahe Campus: No. 4, Section 2, North Jianshe Road, 611731 Chengdu, China; School of Computer Science, Chengdu University of Information Technology, No. 24 Block 1, Xuefu Road, 610225 Chengdu, China

**Keywords:** scRNA-seq, cell type annotation, long-tail distribution, dynamic clustering, continual learning, open-world cell recognition

## Abstract

The rapid accumulation of single-cell RNA sequencing data has provided unprecedented computational resources for cell type annotation, significantly advancing our understanding of cellular heterogeneity. Leveraging gene expression profiles derived from transcriptomic data, researchers can accurately infer cell types, sparking the development of numerous innovative annotation methods. These methods utilize a range of strategies, including marker genes, correlation-based matching, and supervised learning, to classify cell types. In this review, we systematically examine these annotation approaches based on transcriptomics-specific gene expression profiles and provide a comprehensive comparison and categorization of these methods. Furthermore, we focus on the main challenges in the annotation process, especially the long-tail distribution problem arising from data imbalance in rare cell types. We discuss the potential of deep learning techniques to address these issues and enhance model capability in recognizing novel cell types within an open-world framework.

## Introduction

**Figure 1:**
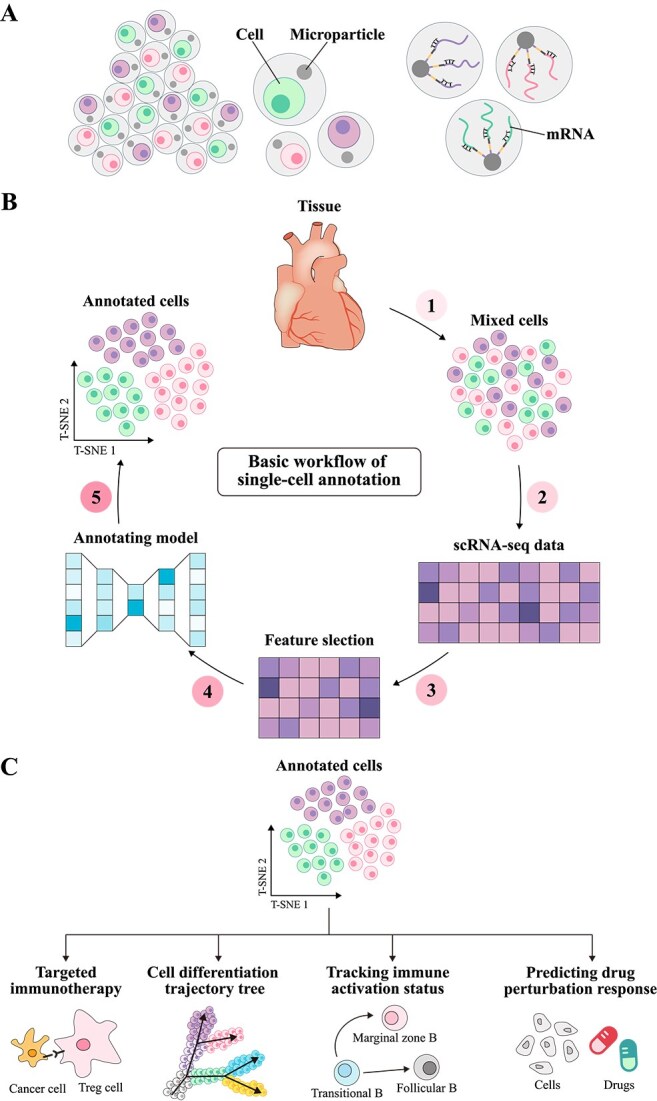
Principles of single-cell annotation. (A) Shows the mRNA extracted from scRNA-seq. As the transcriptional product of cells, mRNA reflects the heterogeneity of gene expression and provides important information for cell type annotation and gene function research. (B) Illustrates the basic workflow of single-cell type annotation. Cells are first extracted from tissue, and single-cell sequencing is performed to obtain a gene expression matrix. High-variance genes are then selected for feature selection. Next, annotation models are used to predict cell types, and the annotation results are finally visualized using dimensionality reduction algorithms such as T-SNE. (C) Demonstrates the applications of single-cell type annotation in various fields, including targeted therapy strategies in the immune microenvironment, cell developmental trajectory reconstruction in developmental biology, immune cell activation state tracking in immunology, and drug perturbation response prediction in precision medicine.

Single-cell type annotation plays a critically prospective role across various research areas within the biomedical field [1, 2]. Although traditional wet-lab approaches, such as immunohistochemistry and fluorescence-activated cell sorting, are reliable, their lengthy development cycles and high costs pose significant challenges for single-cell annotation research [3, 4]. In contrast, single-cell RNA sequencing (scRNA-seq) technology [5] can precisely capture the high variability in gene expression across single cells in the transcriptome by analyzing mRNA levels in individual cells [6, 7] (as illustrated in Fig. 1A). Based on these gene expression data, computational methods can effectively identify and differentiate between various cell types and states [8], revealing their specific functions within complex tissues [9]. This computational approach offers unprecedented potential for exploring cell population heterogeneity and achieving precise annotation.

In recent years, computational annotation methods have demonstrated high accuracy across extensive gene expression profile datasets [10–13], significantly enhancing the efficiency and reliability of annotation processes (the process as shown in Fig. 1B). Depending on the specific applications of transcriptomic gene expression data, current computational methods can generally be classified into four categories. (i) Specific gene expression-based methods employ known marker gene information to manually label cells by identifying the characteristic gene expression patterns of specific cell types [14]. (ii) Reference-based correlation methods categorize unknown cells into corresponding known cell types based on the similarity of gene expression patterns to those in a preconstructed reference library [15]. (iii) Data-driven reference methods predict cell types by training classification models on pre-labeled cell type datasets [16]. (iv) Large-scale pretraining-based methods use large-scale unsupervised learning to capture the deep relationships between cell types by studying generic cell features and gene expression patterns [17].

Several important reviews have systematically examined the development and application of computational methods for single-cell type annotation modeling. Pasquini *et al*. [18] conducted an in-depth analysis of early annotation methods for scRNA-seq data, with a focus on the evolution of automated annotation strategies, including marker gene databases, correlation analysis, and supervised classification, and their applications in cell type identification. The methods reviewed by Pasquini *et al*. [18] established the foundational framework for single-cell data analysis, providing theoretical and technical support for further methodological improvements. Similarly, Cheng *et al*. [19] comprehensively summarized annotation methods based on gene signatures, the application of feature databases, and the crucial role of supervised learning in automated cell type annotation. Their discussion included techniques for improving annotation accuracy through marker gene databases and scoring methods, as well as an analysis of the application of supervised learning in feature selection to optimize model performance and enhance interpretability. These reviews have provided an overview of the current landscape of single-cell type annotation from the perspective of automated annotation strategies and model applications. However, they primarily focus on the frameworks and applications of early methods, with limited discussion of emerging deep learning models, especially in addressing generalization over long-tail distributions [20], open-world data [21], and multi-omics data integration [22]. Consequently, there is a pressing need to integrate the latest computational approaches in single-cell type annotation, delve into the key challenges currently facing the field, and propose potential solutions.

In this work, we provide a comprehensive summary to better understand how to predict single-cell types based on transcriptomic gene features, thereby supporting subsequent single-cell analyses (as shown in Fig. 1C). First, we introduce the existing computational methods for single-cell type annotation, outline the contexts in which each method is applicable, and summarize their primary limitations. Following this, we provide an overview of biological databases used for single-cell type annotation and the processing workflows for scRNA-seq data. Building on this foundation, we explore the key challenges faced by current research and propose potential opportunities to advance single-cell type annotation studies.

## Characteristics and challenges of single-cell transcriptomic data

The accumulation of large-scale single-cell transcriptome data has laid the foundation for the rapid development of cell type annotation methods [23]. Marker gene databases, such as PanglaoDB [24] and CellMarker [25], played a crucial role in the early stages by assisting in the identification of known cell types. However, as research progressed, single-cell gene expression profiles, with their comprehensive depiction of cellular heterogeneity, gradually became the core element of annotation models. The combination of marker genes and gene expression profiles has continuously driven the advancement of annotation technologies [26, 27]. Table 1 summarizes the commonly used public databases, which provide vital support for innovation and future exploration in the single-cell field.

**Table 1:** Comprehensive databases for cellular and transcriptomic research.

**Database**	**Data type**	**Species**	**Info**	**Tissues/cell types**	**Ref**
HCA	Single cell RNAseq	Human	Multi-organ datasets	33 organs	[28]
MCA	Single cell RNAseq	Mouse	Multi-organ dataset	98 major cell types	[29]
Tabula Muris	Single cell RNAseq	Mouse	Multi-organ datasets	20 organs and tissues	[30]
Allen Brain Atlas	Single nuclei RNAseq	Human and mouse	Brain datasets	69 neuronal cell types	[31]
CellMaker 2.0	Marker genes	Human and mouse	Marker database	467 (human), 389 (mouse)	[32]
PanglaoDB	Marker genes	Human	Marker database	155 cell types	[24]
CancerSEA	Marker genes	Human cancer	Marker database	14 cancer functional states	[33]
Immune Cell Atlas	Single cell RNAseq	Human	Immune cell datasets	Immune system cells	[34]
Human Cell Landscape	Single cell RNAseq	Human	Human atlas of immune cells	Immune cells across tissues	[35]
GEO	RNAseq, microarray	Human, mouse, various	Gene expression profiles	Multiple organs and tissues	[36]
GTEx	RNAseq, genomics	Human	Tissue-specific gene expression	54 tissues	[37]

### Impact of sequencing platforms on cell type annotation

The rapid advancement of scRNA-seq has provided a powerful tool for dissecting cellular heterogeneity, state transitions, and their roles in complex biological processes. At its core, scRNA-seq involves extracting mRNA from individual cells, reverse-transcribing it into cDNA, and obtaining gene expression profiles of single cells through high-throughput sequencing. Compared to traditional bulk RNA-seq, scRNA-seq can resolve subtle differences in gene expression at the single-cell level, enabling precise characterization of cell types, developmental states, and dynamic changes during specific biological processes. This high-resolution sequencing technology has played a crucial role in fields such as tumor microenvironments, immune cell populations, and developmental biology.

Despite the significant advancements in scRNA-seq technology that have enhanced cell type annotation capabilities, differences among sequencing platforms have profoundly impacted annotation outcomes. Various platforms, such as 10x Genomics and Smart-seq, exhibit distinct data characteristics due to differences in their sequencing principles. For instance, 10x Genomics [38] relies on droplet-based encapsulation for high-throughput sequencing, enabling rapid profiling of large cell populations but often resulting in higher data sparsity. In contrast, Smart-seq [39] employs a full-transcriptome amplification strategy, detecting more genes with higher sensitivity, which aids in identifying rare transcripts. However, these technical differences worsen key challenges in scRNA-seq: sparsity, heterogeneity, and batch effects. In cross-platform applications, these factors frequently result in inconsistent annotation performance.

Specifically, the lower gene detection rate of the 10x Genomics platform may hinder the model’s ability to capture key marker genes of rare cell types, while the Smart-seq platform, capable of detecting more genes, may reveal finer-grained cell subpopulations that exceed the classification capacity of pre-trained models. Additionally, differences in sequencing depth, primer bias, and other factors often result in significant batch effects across platforms, compromising the comparability of gene expression profiles. Without effective preprocessing strategies, such as batch correction or cross-platform normalization, these systemic biases can directly undermine the model’s generalization ability. Collectively, these issues contribute to the reduced stability of existing annotation models in diverse data environments, representing one of the core challenges in scRNA-seq data analysis.

### Dynamic updates and sustainability of marker gene

Marker genes play a central role in single-cell research, with their specific expression significantly enhancing the accuracy of cell type annotation and functional analysis. For example, CD133, as a stem cell marker [40], is widely used in stem cell identification and behavioral studies [41, 42], while CD3 [43] and CD19 [44] are classical markers for T cells and B cells, respectively, forming the foundation for the classification and functional analysis of immune cells. These marker genes, through stable and specific expression, provide researchers with a quick and reliable means of analyzing complex cell populations. However, existing marker gene databases, such as CancerSEA [33], CellMarker 2.0 [32], and PanglaoDB [24], have notable limitations, including the absence of certain marker genes, outdated data, and a lack of consistency across samples, which restrict their performance in handling novel cell types or rare cell populations.

In recent years, the introduction of deep learning technologies, such as the self-attention mechanisms of Transformer [45] models, has shown significant advantages in gene selection and feature discovery. For instance, methods like SCTrans [46] leverage attention mechanisms to capture gene combinations that are frequently focused on in gene expression profiles, identifying specific genes highly consistent with marker gene databases and expanding the understanding of previously unseen cell types. This approach not only compensates for the shortcomings of marker gene databases but also provides a powerful tool for discovering new marker genes in an open-world context. In the future, combining the automatic feature selection capabilities of deep learning models with biological validation from experts will enable the dynamic updating of marker gene databases, thereby continuously improving their utility and accuracy in single-cell annotation. This direction will provide essential support for identifying unknown cell types and analyzing complex cellular heterogeneity.

### Data preprocessing before annotation

**Figure 2:**
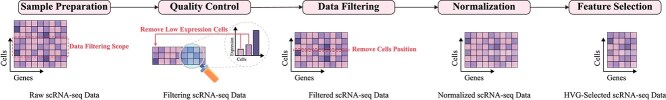
Data preprocessing workflow for single-cell type annotation. Sample data undergo quality control to identify and remove cells with low expression or those requiring exclusion for other reasons. Subsequently, the remaining cell data is subjected to log normalization, and a specific number of highly variable genes are selected based on task requirements, completing the core steps of data preprocessing.

The preprocessing pipeline in single-cell data analysis forms the foundation for ensuring the accuracy of cell type annotation. First, quality control (QC) is performed by evaluating metrics such as the number of detected genes [47], total molecule count, and the proportion of mitochondrial gene expression, thereby eliminating low-quality cells and technical artifacts. Data filtering further refines the dataset by removing noise samples, such as doublets or high-noise cells, thereby improving data quality [48]. Next, normalization removes technical biases, ensuring that gene expression levels are comparable across different cells, thus enabling cross-sample analysis for annotation models [49]. Finally, feature selection identifies highly variable genes (HVGs), highlighting gene expression signals relevant to cell-type specificity and providing essential inputs for capturing biological heterogeneity in models [50]. Figure 2 illustrates this systematic preprocessing workflow, emphasizing the critical role of each step in enhancing single-cell annotation accuracy.

### Batch effect correction methods

The sparsity of scRNA-seq data primarily arises from both technical noise, such as low mRNA capture efficiency, and biological factors, including the absence of low-abundance transcripts. This results in a high proportion of zero values in the gene expression matrix, which interferes with the identification of rare cell types and weakens the accuracy of gene co-expression network construction. To address this issue, researchers have proposed multi-level solutions. SCTransform [51] corrects technical biases by modeling the mean-variance relationship of gene expression, effectively reducing the influence of sequencing depth on data quality. Discriminative component analysis (DCA) [52] mitigates data sparsity by leveraging intercellular expression similarity to impute missing values, thereby improving the detection of rare cell types. Additionally, dimensionality reduction methods like PHATE [53] enhance the topological structure of the data, optimizing cell trajectory inference.

Beyond sparsity, the high heterogeneity and batch effects in scRNA-seq data present fundamental analytical challenges. Differences in sequencing platforms, such as the droplet-based 10x Genomics and the full-transcriptome Smart-seq, introduce significant platform-specific variations that exacerbate data heterogeneity. Further discrepancies arise from variations in experimental batches, sample sources, and sequencing depth, leading to batch effects that complicate the direct integration of scRNA-seq datasets from different experiments.

To address these challenges, researchers have developed various cross-batch integration strategies. Mutual nearest neighbors (MNN) [54] constructs a linear mapping model by pairing cells across datasets to eliminate nonlinear shifts, making it particularly effective for small-scale batch differences. Harmony [55] applies iterative soft clustering and latent space alignment to remove systematic technical biases while preserving biologically meaningful variation. The Seurat [56] integration tool utilizes canonical correlation analysis (CCA) to identify dataset-wide anchors and employs a shared nearest neighbor (SNN) graph to achieve robust integration of high-dimensional sparse data [57].

Experimental results indicate that combining these methods, such as applying SCTransform for normalization before integrating data with Harmony, significantly improves data retention, enhances the resolution of downstream clustering, and effectively mitigates batch shifts across platforms. However, excessive imputation may introduce spurious associations, underscoring the need for cross-validation strategies, such as holding out a gene validation set, to strike a balance between data completeness and biological authenticity.

## Methods of single-cell type annotation

**Figure 3:**
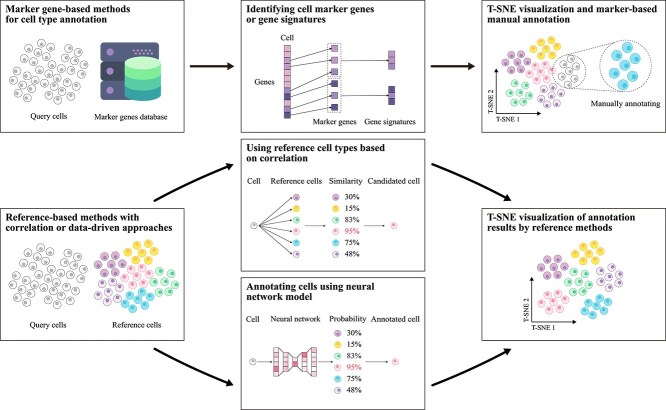
Flowchart of single-cell type annotation methods. This chart depicts two main workflows: one using specific gene databases and the other referencing annotated cell-type samples. The specific gene-based method clusters cells and uses marker genes for annotation, while the reference-based method matches cell data to reference databases via correlation or data-driven models. Results are visualized with dimensionality reduction techniques like t-SNE.

Single-cell type annotation plays a crucial role in unraveling cellular heterogeneity and advancing single-cell analysis [58, 59]. With the continuous advancement of computational methods, annotation approaches have diversified, resulting in several primary strategies [18, 60]. Currently, these methods can be categorized into four major types. In the following sections, we will discuss the representative models within each category in detail, examining the specific problems they address, their applicable contexts, and their respective strengths and limitations. Furthermore, we have consolidated these four methods into the two annotation workflows illustrated in Fig. 3: one relying on specific gene databases as advisory resources, and the other leveraging previously annotated cell type samples as references.

### Methods based on specific gene expression

**Figure 4:**
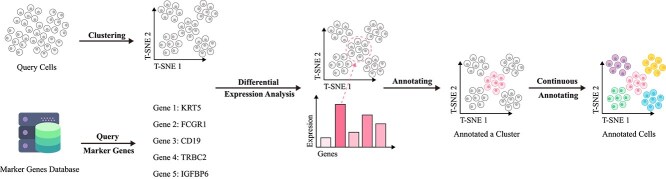
Basic workflow of annotation methods based on specific gene expression. Cell samples are first clustered using a clustering algorithm, then specific cell types within each cluster are identified by querying differential expression genes from biomarker databases.

In single-cell transcriptomics, specific gene markers are categorized into marker genes and gene signatures [61, 62]. Accordingly, cell annotation methods based on specific gene expression can be classified into two distinct approaches. The marker gene approach relies on the specific expression of a single gene within a particular cell type, typically used for rapid differentiation of well-defined cell types [63, 64]. In contrast, the gene signature approach identifies a set of genes co-expressed within a given cell type, offering a more comprehensive characterization of cell features [65, 66]. This method is particularly advantageous for the identification of cell subtypes and low-abundance cell populations. The standard schematic of these methods is presented in Fig. 4. Table 2 and the subsequent sections provide a detailed overview of these techniques.

**Table 2:** Techniques for single-cell type annotation methods based on specific gene expression, including their key algorithm, programming language, and feature and input characteristics.

**Model**	**Year**	**Key algorithm**	**Language**	**Feature and input characteristics**
scSorter [68]	2021	Marker gene scoring	R	Focuses on marker gene expression, tailored for scRNA-seq data
SCINA [76]	2019	Expectation-maximization (EM), bimodal distribution	R	Marker gene scoring with bimodal distribution, designed for scRNA-seq
Seurat v3 [56]	2019	kNN graph, transfer learning	R	Integrates multi-omics data (scRNA-seq, scATAC-seq), supports cross-platform fusion
ScType [64]	2022	Louvain clustering	R	Focused on cancer cell annotation, optimized for scRNA-seq datasets
CellID [77]	2021	SVD, multiple correspondence analysis (MCA)	R, C++	Gene signature identification, applies to scRNA-seq
CellAssign [14]	2019	Bayesian inference for marker-based classification	R	Marker-based probabilistic classification, suitable for scRNA-seq
scCATCH [79]	2020	Evidence-based marker scoring	R	Focuses on evidence-weighted marker gene prioritization, suitable for scRNA-seq

#### Marker gene-based methods

Marker gene-based cell annotation methods typically combine unlabeled data with partially annotated information to accommodate dataset complexity. These methods leverage gene expression patterns for precise cell type identification but face challenges with complex cell populations where cellular subtypes show minimal differences or high data noise [2, 67]. In open world settings, where new cell types lacking marker genes emerge, the identification accuracy of traditional methods declines [68]. Rare cell types with long-tail distributions are also prone to being overlooked in the annotation process.

To address these challenges, a series of improved methods have emerged in recent years, which can be categorized into clustering-based methods, such as Seurat [69], and probabilistic model-based methods [70, 71], including CellAssign [14] and scSorter [68]. Among these, Seurat, which annotates cell types based on clustering and marker genes, remains the most reliable approach. Seurat first normalizes and performs dimensionality reduction (e.g. PCA, UMAP) on single-cell data, followed by clustering analysis to group cells. It then identifies marker genes for each cluster through differential expression analysis and compares them with known marker genes. By integrating prior biological knowledge, Seurat ultimately assigns cell types to each cluster. CellAssign combines a Bayesian probabilistic model with the expectation-maximization (EM) algorithm [72] to compute the posterior probability of each cell belonging to different cell types based on a predefined set of marker genes, thereby enabling cell type assignment. Additionally, it supports an “unassigned” state [73], allowing the model to recognize novel cell types that may not be included in the predefined marker gene list, making it suitable for large-scale and complex datasets. However, its performance may be limited when marker genes are missing, expression noise is high, or when dealing with rare and previously unseen cell types. scSorter constructs a semi-supervised classification framework, leveraging both marker and non-marker gene expression information to enhance classification robustness. While maintaining marker gene guidance, scSorter also incorporates auxiliary information from non-marker genes, improving its ability to classify cells. In particular, when marker gene expression is low or data sparsity is high, scSorter remains effective in capturing cell type characteristics and enhances the identification of lowly expressed marker genes.

While these methods advance complex cell type annotation, further improvement is needed to effectively address challenges with new cell types in open-world contexts, rare cell types, and incomplete marker genes. Future research may focus on expanding marker gene databases and developing more robust algorithms to tackle these challenges.

#### Gene signature-based methods

Gene signature-based cell annotation methods are an evolution of traditional marker gene approaches, aiming to overcome limitations associated with relying on a single specific gene [74, 75]. By integrating a group of co-expressed genes, gene signature methods provide a more comprehensive cellular profile, enabling more accurate annotation of complex cell types and their subtypes. SCINA [76] and CellID [77] exemplify leading strategies in this area. SCINA employs a semi-supervised algorithm that combines gene signatures with an EM strategy [72], effectively enhancing the detection of distinct cellular characteristics and excelling in annotating low-abundance cell types. CellID, on the other hand, uses multiple correspondence analysis (MCA) [78] for dimensionality reduction, preserving the diversity of gene expression patterns and achieving greater stability and consistency across various experimental conditions and parameter settings, which is especially important for cross-dataset analysis. Despite the improved annotation accuracy brought about by enhanced gene feature detection, gene signature methods still encounter critical challenges. On one hand, in identifying rare cell types with long-tail distributions, gene expression heterogeneity can limit their performance. In open-world settings lacking known gene combinations, gene signature methods, being similar to traditional marker gene approaches, demonstrate limited adaptability for recognizing unknown cell types. Overall, future research should focus on optimizing these methods by addressing data heterogeneity to develop more precise and broadly applicable annotation approaches.

### Methods based on reference and correlation analysis

**Figure 5:**
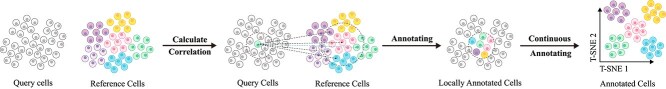
Basic workflow of reference-based annotation methods using correlation. The process begins by establishing correlation relationships between the query cells to be annotated and the reference cell samples. The most similar reference cells are then selected as the basis for determining the cell types of the query cells. This workflow is subsequently extended to annotate all query cell samples.

The correlation-based reference methods for cell type annotation infer cell types by evaluating gene expression similarities between target cells and known reference datasets (refer to Table 3). These methods are generally categorized into two strategies: single-cell similarity analysis and centroid-based similarity analysis. The former is ideal for high-resolution single-cell annotation, while the latter is better suited to large-scale cell population analysis. Common similarity metrics, including Pearson correlation coefficient [80], Spearman rank correlation coefficient [81], and cosine similarity [82], offer precise quantification of expression profile similarities across cells. Figure 5 provides an intuitive and illustrative representation of the basic workflow of reference-based annotation methods using correlation.

**Table 3:** Techniques for single-cell type annotation models based on correlation methods, including their approach, programming language, and key descriptions.

**Model**	**Year**	**Technology**	**Language**	**Description**
CHETAH [10]	2019	Classification Tree	R	Uses a classification tree for annotation, identifying novel cell types.
SingleR [15]	2019	Spearman correlation	R	Calculates Spearman correlation for matching.
scamp [11]	2018	Correlation, KNN	R	Combines cosine, Spearman, and Pearson correlation with KNN.
Cell BLAST [84]	2020	GAN	Python	Employs a generative adversarial network for low-dimensional embeddings and unseen cell identification.
scMatch [86]	2022	Correlation	Python	Leverages Spearman and Pearson correlation for large-scale datasets.
scLearn [90]	2020	DCA	R	Uses discriminative component analysis with automatic threshold selection.
ClustifyR [91]	2020	Correlation	R	Integrates multiple data sources with Spearman, Pearson, Kendall, and cosine correlation.

Early correlation-based reference tools like scmap [11] used K-nearest neighbor (KNN) [83] algorithms to match cell types, annotating based on similarity measures. However, when dealing with complex and highly heterogeneous tumor samples, these methods faced considerable uncertainty. To address this limitation, improved tools have been developed. For instance, CHETAH [10] employs a hierarchical classification tree to progressively match cell types, enhancing its capacity to analyze high-heterogeneity samples, particularly in tumor classification. Cell BLAST [84], on the other hand, introduces generative adversarial networks (GANs) [85] to dynamically adjust the model to new data, demonstrating strong adaptability in multi-source data integration scenarios. scMatch [86] tackles the challenge of annotating low-coverage scRNA-seq data by computing gene expression similarity with large reference datasets like FANTOM5 [87], thus improving robustness to high-dimensional sparse data.

Although these methods represent a significant improvement over traditional marker gene-dependent models, avoiding the limitations of over-relying on databases, and making progress in multi-source data integration and high heterogeneity data classification, they still face several challenges. Specifically, the generalization ability of current methods remains insufficient, particularly in handling batch effects across sequencing standards and species. Therefore, future research could focus on incorporating the concept of continual learning [88, 89], expanding the available scRNA-seq datasets for reference, and enhancing the generalization and continual learning capabilities of correlation-based reference methods.

### Methods based on data-driven references

**Figure 6:**
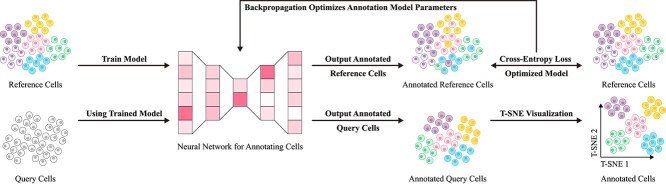
Basic workflow of data-driven reference methods. First, reference data with well-annotated labels are input into a neural network model for training, enabling the model to learn to identify cell types based on gene expression differences under a supervised learning paradigm. Next, query cell data are fed into the trained neural network model to achieve precise cell type annotation.

Data-driven methods leverage extensive datasets to enable machine learning models to automatically extract features for cell type annotation. In contrast to specific gene expression and correlation-based reference methods, data-driven approaches offer superior flexibility, capable of autonomously uncovering complex patterns within data. This adaptability effectively addresses limitations of traditional methods in capturing cellular diversity and complexity [17]. Conventional approaches rely heavily on manually selected marker genes or predefined reference sets, making it challenging to comprehensively represent high-dimensional data, often leading to the omission of rare cell types [92]. By contrast, data-driven methods achieve substantial gains in annotation accuracy and generalizability through deep feature extraction [93]. Figure 6 illustrates the basic implementation workflow of such methods, while Table 4 present the advantages and applicability of these methods across various implementation strategies.

**Table 4:** Techniques for single-cell type annotation models based on data-driven reference methods, including their approach, programming language, characteristics, and learning types.

**Model**	**Year**	**Technology**	**Language**	**Characteristics**	**Learning Type**
mtANN [16]	2023	AE, Ensemble model	Python	Ensemble learning, multi-model approach	Supervised learning
scMMT [117]	2024	Convolutional neural network (CNN)	Python	CITE-seq, scRNA-seq, protein prediction	Supervised learning
scMGCN [118]	2024	Graph convolutional network (GCN)	Python	Multi-view learning, single-cell data integration	Semi-supervised learning
scSemiCluster [103]	2020	Deep clustering algorithm	Python	Structural regularization, clustering	Semi-supervised learning
TOSICA [13]	2023	Transformer	Python	Transformer architecture, interpretable annotation	Supervised learning
CAMLU [119]	2022	AE, support vector machine (SVM)	R	Iterative feature selection, novel cell identification	Supervised learning
scAnno [120]	2023	Deconvolution	R	Supervised classification, cell type identification	Supervised learning
scDeepSort [102]	2021	Graph neural network (GNN)	Python	Pre-trained model, weighted GNN	Supervised learning
scTransSort [105]	2023	Transformer, CNN	Python	Gene expression embeddings, data sparsity reduction	Supervised learning
TripletCell [121]	2023	k-nearest neighbors (KNN)	Python	Deep metric learning, triplet loss	Supervised learning
CALLR [122]	2021	Laplacian, logistic regression	R	Graph Laplacian, sparse logistic regression	Supervised learning
scGAD [107]	2023	K-means	Python	Generalized annotation, clustering labels	Unsupervised learning
SciBet [12]	2020	Multinomial distribution model	R, C++	Multinomial distribution, maximum likelihood estimation	Supervised learning
scDeepInsight [123]	2023	CNN	Python	Image transformation, supervised annotation, data integration	Supervised learning
CIForm [106]	2023	Transformer	Python	Transformer, patch concept, computational complexity reduction	Supervised learning
scPred [96]	2019	SVM	R	Unbiased feature selection, probabilistic machine learning	Supervised learning
ItClust [124]	2021	Confidence score	Python	Iterative transfer learning, fine-tuning	Transfer learning
scGCN [125]	2021	GCN, mutual nearest neighbors (MNN)	Python	Semi-supervised GCN, mixed graph	Semi-supervised learning
scNym [126]	2021	Generative adversarial network (GAN)	Python	Adversarial training, pseudo-labels	Unsupervised learning
ACTINN [127]	2020	Artificial neural network (ANN)	Python	Minimal prior knowledge, flexible learning	Supervised learning
SingleCellNet [97]	2019	Random forest (RF)	R	Top-pair transformation, discriminative gene pairs	Supervised learning
scArches [109]	2022	Variational autoencoder (VAE)	Python	Transfer learning, efficient construction	Transfer learning
scNAME [128]	2022	K-means	Python	Contrastive learning, neighborhood-based methods	Unsupervised learning
scLearn [90]	2020	Discriminative component analysis (DCA)	R	Threshold selection, novel cell identification	Supervised learning
SC3 [129]	2017	K-means	R	Gene filtering, consensus clustering	Unsupervised learning
scziDesk [130]	2020	AE, soft K-means	Python	Denoising autoencoders, soft K-means, clustering	Unsupervised learning
SCTrans [46]	2024	Transformer	Python	Multi-scale Transformer, gene sub-vectors	Supervised learning
scEvolve [113]	2024	Prototypical contrastive replay	Python	Forgetting mitigation, memory buffer	Continual learning
scTab [131]	2024	Transformer	Python	Feature attention, data augmentation	Supervised learning
scPOT [111]	2023	Optimal transport (OT)	Not found	Novel type discovery, automatic cell type count estimation	Supervised learning
scDET [132]	2024	AE, K-means	Not found	Distribution-independent framework, contrastive learning, long-tail identification	Unsupervised learning

In the early stages of single-cell annotation research, traditional machine learning methods, such as support vector machines (SVM) [94] and random forests (RF) [95], were widely applied. For instance, representative methods like scPred [96] and SingleCellNet [97] utilized SVM and RF classifiers to analyze gene expression data. Compared to approaches based on marker genes and correlation, these machine learning strategies exhibited greater flexibility and efficiency. By leveraging supervised learning to extract features from annotated data, these methods effectively mitigated noise and sparsity in gene expression data to some extent, demonstrating strong performance on early single-cell datasets. However, their ability to handle data sparsity heavily relied on feature engineering, particularly the selection of highly variable genes (HVGs). In 2017, McCarthy *et al*. [98] proposed a standard HVG selection procedure that retained genes with the highest coefficient of variation across cells, typically comprising 10%–20% of all genes. This approach efficiently filtered out low-information loci, reducing the feature dimensionality of RF classifiers by 80%–90% while maintaining over 90% classification accuracy on normalized datasets [99]. This strategy was later adopted by deep learning methods, such as single-cell variational inference (scVI) [100], where the encoder preferentially processed the HVG subset. Although HVG selection alleviated certain sparsity issues, the increasing throughput of single-cell sequencing has introduced new challenges, particularly the issue of zero inflation in cross-platform data integration. For example, in T-cell subtype classification, training on mixed datasets from 10x Genomics and Smart-seq2 platforms resulted in a 15%–22% decline in the recall rate of SVM [101]. Furthermore, these methods have gradually revealed limitations in batch effect correction, adaptation to cross-dataset distribution shifts, and the identification of rare cell types.

To overcome these challenges, the advent of deep learning has driven substantial advancements in single-cell type annotation. Deep learning enables automatic feature extraction, addressing traditional machine learning methods’ deficiencies in batch effect control and generalization. For example, scDeepSort [102] employs a weighted graph neural network to handle complex inter-data relationships, significantly enhancing annotation accuracy without the need for additional reference data. Similarly, scSemiCluster [103] utilizes semi-supervised learning and structural similarity regularization to further mitigate batch effect issues and improve adaptability to diverse datasets. However, while deep learning has advanced generalization performance, it still faces challenges in capturing rare cell types within long-tail distributions [104]. These models often exhibit a tendency to focus on mainstream features in the data, with limited attention to the feature expressions of rare types.

Addressing this, Transformer [45] models have gradually entered the field of single-cell annotation, offering new strategies to address the challenges of rare cell types within long-tail distributions. The self-attention mechanism of Transformers allows them to flexibly focus on critical features in the data, making them particularly suited for capturing the feature expressions of rare cell types. For example, mtANN [16] and TOSICA [13] integrate self-attention mechanisms with multi-gene selection strategies, significantly enhancing the recognition of rare cell types. scTransSort [105] further optimizes sparse data handling, enabling the model to extract more comprehensive feature representations, thereby improving annotation efficiency and robustness. Additionally, CIForm [106] introduces a “patch” concept, effectively reducing computational complexity, thus providing new methods for large-scale single-cell data analysis. Overall, the Transformer architecture not only strengthens long-tail distribution recognition but also enhances accuracy in cell annotation tasks.

Beyond the issue of long-tail distributions, single-cell annotation must also contend with the challenge of identifying unknown cell types in an open-world context. In response, semi-supervised and unsupervised learning strategies are being explored. scGAD [107] uses K-means [108] clustering to generalize potential unknown cell types, allowing the model to distinguish new cell types rather than merely labeling them as “unassigned.” Moreover, scArches [109] combines variational autoencoders (VAE) [110] with transfer learning to generate cross-platform reference maps, further enhancing model generalizability across different data platforms. In addition, scPOT [111] employs an optimal transport (OT) [112] framework to accurately annotate and identify unknown cell types, providing an innovative solution for rare type recognition within open sets.

Meanwhile, data-driven methods often exhibit limited flexibility when applied to unseen or external datasets. These methods are susceptible to overfitting the training data, making it difficult to maintain stable and high performance on novel datasets. In contrast, unsupervised approaches based on marker genes or gene signatures typically demonstrate greater robustness and adaptability when processing new data. With the integration of continual learning into the single-cell field, scEvolve [113] represents the first model to achieve single-cell incremental learning [114] and improve predictive generalization through data replay. Extensive evaluations on a series of rigorously curated benchmark datasets consistently demonstrate that scEvolve can continuously assimilate scRNA-seq data from different batches and sequencing platforms over prolonged periods, effectively identifying diverse cell types across various tissues. Furthermore, it alleviates the overfitting risks and generalization limitations inherent to data-driven methods while mitigating catastrophic forgetting when incorporating new datasets. Thus, continual learning provides a promising avenue for advancing data-driven methodologies, fostering enhanced flexibility and superior generalization capabilities.

Despite significant improvements in annotation accuracy and generalizability, data-driven methods’ reliance on data quality still poses a risk of information loss. Future directions include integrating multi-omics data to address information gaps, leveraging self-supervised learning [115] to maximize the utility of unlabeled data, applying knowledge distillation [116] to facilitate cross-model knowledge transfer, and adopting continual learning to enhance model adaptability to new data. These advancements aim to provide richer contextual information for single-cell annotation, further improving model adaptability and accuracy, and delivering more comprehensive and flexible solutions for cell type identification.

### Methods based on large-scale pretraining

**Figure 7:**
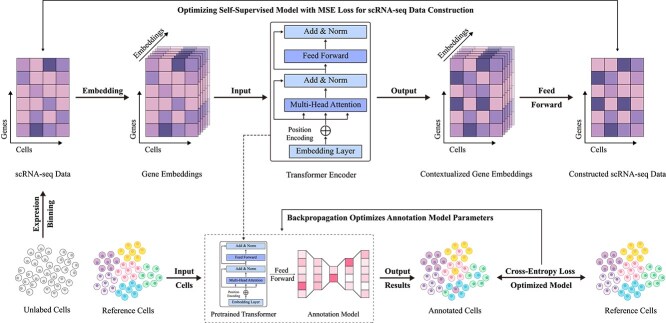
Basic workflow of large-scale pretraining methods. This approach begins by extracting scRNA-seq data from large-scale unlabeled single-cell samples as a comprehensive feature foundation. Using gene embeddings, an encoding-decoding strategy is employed to reconstruct scRNA-seq data in a self-supervised learning framework, while simultaneously pretraining a Transformer encoder as a deep feature extraction model. The pretrained model is then applied to cell type annotation tasks under a data-driven supervised learning paradigm.

To address the common issue of information loss in traditional machine learning methods, large-scale pretraining approaches have emerged as an effective solution [115]. These methods leverage self-supervised learning to extract underlying gene expression patterns and cellular features from vast amounts of unlabeled data, effectively reducing the information loss typically encountered in high-dimensional data processing. By capturing complex relationships and latent structures within the data without requiring manual labeling, self-supervised learning not only compensates for missing information but also significantly improves model generalization, enabling the identification of a broader range of complex cellular characteristics (as detailed in Table 5). The basic workflow of this approach is illustrated in Fig. 7.

**Table 5:** Techniques for single-cell type annotation models based on large-scale pretraining methods, including their approach, programming language, parameter size, input modality, multi-task capabilities, and explainability.

**Model**	**Year**	**Technology**	**Language**	**Parameters (Estimation)**	**Input Modality**	**Multi-task (Tasks)**	**Explainability (From papers)**
scBERT [17]	2022	BERT	Python	5M	scRNA-seq	No	Yes, attention weights for gene relevance, identifying key genes for cell types
scGPT [133]	2024	GPT	Python	38M	Multi-omics (scRNA-seq, scATAC-seq, protein)	Yes, annotation, perturbation analysis, multi-batch integration	Yes, gene pathway interpretation via latent features, identifying gene interactions
scFoundation [134]	2024	Transformer	Python	100M	scRNA-seq	Yes, annotation, clustering, drug response prediction	No, but provides cell and gene embeddings for downstream analysis
scRobust [135]	2024	Transformer	Python	18M	scRNA-seq or scATAC-seq	Yes, annotation, drug tolerance, scATAC-seq analysis	Yes, maximizing the highly unique genes of each cell

In recent years, several large-scale pretrained models for single-cell annotation, such as scBERT [17], scGPT [133], and scFoundation [134], have made remarkable advances. Through self-supervised learning, these models extract gene expression patterns and cellular features from large-scale unlabeled data, effectively overcoming the limitations of traditional methods with respect to information loss. A primary advantage of these approaches lies in their reliance on extensive unlabeled data for pretraining [136], which allows models to automatically capture deep structures within data and learn more intricate cellular features, thus enhancing cell type recognition capabilities and circumventing information loss caused by high data dimensionality or limited labeling. Moreover, research shows that larger model parameters often yield better performance, as increased model capacity enables richer feature extraction. Additionally, large-scale pretrained models exhibit strong transferability, demonstrating robustness and adaptability across various tasks and datasets, thereby advancing the field of single-cell annotation.

Although large-scale pretraining methods have made significant advances in improving annotation accuracy and generalization, they still face several challenges. First, these methods require high-quality data [137] and substantial computational resources, particularly when handling large-scale datasets. Second, their generalization remains limited [138] when applied to highly heterogeneous or noisy data, especially across different biological conditions and experimental platforms. Additionally, as model parameters scale up, computational and storage costs increase significantly, restricting their practical feasibility [139]. To address these issues, scRobust introduces strategies such as random gene subset pretraining, multi-task collaborative optimization, a highly unique gene-driven dynamic input mechanism, and a lightweight model architecture. These innovations effectively mitigate the sensitivity of traditional self-supervised methods to data quality, excessive computational demands, and limited cross-platform generalization, providing an efficient and robust solution for single-cell analysis. While large-scale pretraining, which uses self-supervised learning to extract deep gene expression patterns from unlabeled data, reduces information loss in data-driven methods, its limitations in data quality, computational efficiency, and cross-dataset generalization remain unresolved.

## Experimental evaluation of single-cell annotation

### Evaluation metrics

The performance of single-cell annotation models is typically evaluated based on their performance on test data, to assess the model’s applicability to new data. Cross-validation (CV) is commonly used for model evaluation [140], where the data are split into training and test sets. The training data are used for model learning, while the test data are used to assess the model’s performance. $K$-fold cross-validation is a popular method, where the dataset is divided into $K$ equal parts. Each time, one part is selected as the test set, and the remaining $K-1$ parts are used as the training set. This process is repeated $K$ times, with each subset being used as the test set in turn, and the average result from the $K$ tests is taken as the model’s evaluation score. To balance computational efficiency and evaluation quality, $K$ is usually chosen as 5 or 10 [141].

In single-cell type classification tasks, classification performance can be measured using various evaluation metrics, most of which are based on a “confusion matrix” that includes four key elements: True Positives ($TP$), False Positives ($FP$), True Negatives ($TN$), and False Negatives ($FN$). Based on these values, key performance indicators such as $Accuracy$, $Precision$, $Recall$, and $F1-score$ can be calculated. The formulas for these calculations are as follows: 


(1) \begin{align*} & Accuracy=\frac{TP+TN}{TP+FP+FN+TN} \end{align*}

(2) \begin{align*} & Precision=\frac{TP}{TP+FP} \end{align*}

(3) \begin{align*} & Recall=\frac{TP}{TP+FN} \end{align*}

(4) \begin{align*} & F1-Score=2\times\frac{Precision\times Recall}{Precision+Recall} \end{align*}

### Performance evaluation

**Figure 8:**
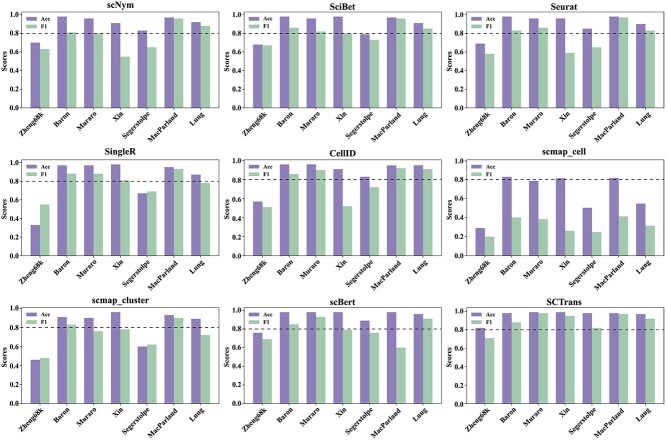
Comparison of annotation performance of different single-cell annotation methods across multiple datasets. The figure presents bar charts evaluating the performance of nine methods on seven benchmark datasets, where a higher bar indicates better performance of the method.

In evaluating the performance of various single-cell annotation methods, we adopted the benchmark results reported by Lin *et al*. [46], conducting a comprehensive analysis of these methods across multiple datasets. Figure 8 presents a comparison of their performance in terms of accuracy and F1 scores. The results indicate that deep learning-based approaches, such as SCTrans [46] and scBERT [17], demonstrate a clear advantage, consistently achieving superior performance across diverse datasets and exhibiting exceptional generalization capabilities. By contrast, traditional methods, including Seurat [69] and the gene signature-based CellID [77], show greater variability in performance, particularly with limited adaptability to cross-dataset scenarios.

**Figure 9:**
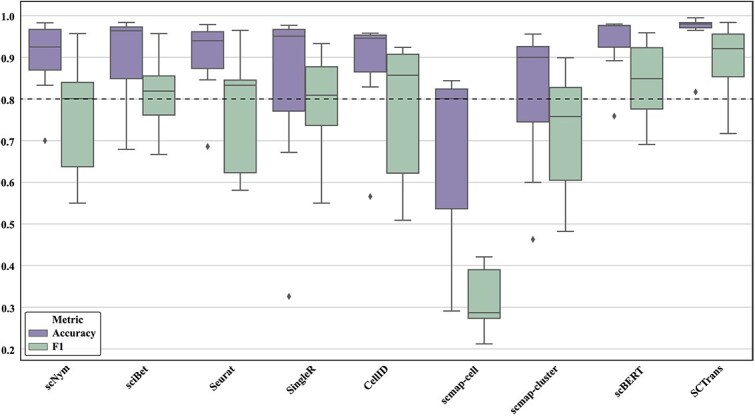
Comparison of stability of different single-cell annotation methods across multiple datasets. The figure shows the stability of each method across different benchmark datasets using boxplots. A higher position and smaller range between the upper and lower quartiles of the boxplot indicate better stability of the method.

The boxplot in Fig. 9 further clarifies this trend, revealing that deep learning models show higher stability across datasets, while traditional methods exhibit greater fluctuation. Overall, deep learning methods outperform traditional computational methods in terms of robustness and generalization across multiple datasets, with the latter showing some advantages on certain datasets but lacking overall stability.

## Challenges and opportunities

Despite significant progress in single-cell type annotation, several pressing challenges remain, primarily including the issue of long-tail distribution in datasets, the ability to generalize to unseen cell types, and the effective annotation of new sequencing datasets using existing models.

### Enhancing single-cell annotation with multi-source data perception

In single-cell type annotation, traditional single-omics methods, due to their reliance on data from a single source, often struggle to fully capture the complex features of cells. For example, scRNA-seq data can reveal transcriptional features of cells but lacks information on other important aspects, such as chromatin accessibility [142] and protein expression [143]. This limitation results in less accurate annotations, particularly for rare cell types or subtypes, especially in tissues with high heterogeneity.

To overcome these limitations, the concept of multi-source perception advocates for the integration of multiple omics data, expanding the model’s understanding of cellular features across multiple layers. By leveraging the complementary advantages of various omics sources, models can capture the relationships between them, thus providing a more comprehensive perspective for cell annotation [144]. Currently, methods like scJoint [145] and TotalVI [146] have made progress in this area. For instance, scJoint integrates scRNA-seq and single-cell assay for Transposase-Accessible Chromatin using sequencing (scATAC-seq) data into a shared latent space, facilitating the combination of different omics data. TotalVI, based on a variational autoencoder [110] model, combines transcriptomics and proteomics, reducing biases from differences in omics technologies. However, constructing robust latent spaces in high-dimensional, sparse multi-omics data and ensuring effective retention of features from all omics remain major challenges for multi-source perception.

Notably, existing studies have systematically demonstrated that multi-omics integration can significantly enhance the annotation performance of rare cells. For instance, CITE-seq technology, which combines transcriptomic and proteomic data, successfully identified $< $0.5% circulating NK cells that were previously missed by single-omics approaches [147]. Similarly, after integrating transcriptomic and epigenomic data, the MOFA+ framework improved the clustering purity of 2% endocrine precursor cells from 68% to 92% [148]. In synthetic data testing, multi-omics approaches increased the recall rate for 1% rare cells by 27% compared to single-omics methods [149], and multimodal integration demonstrated statistically significant advantages in identifying low-abundance cell populations ($< $5%) [57]. These findings suggest that multi-source perception not only expands the feature space but also enhances rare cell resolution through cross-validation of signals across different omics layers.

To further improve the application of multi-source perception in single-cell annotation, strategies such as self-supervised learning [115] and knowledge distillation [116] can be introduced to enhance the model’s deeper understanding of multi-omics features. For example, the multi-source perception process could be simulated in the latent space, allowing the model to adaptively learn core features of cell types from each omic layer, thereby preserving the unique information of each omics dataset during integration. Additionally, evaluating cross-dataset generalization ability could improve the adaptability of multi-omics methods across different experimental conditions and technical platforms, ultimately enhancing annotation accuracy and robustness. Such improvements would help multi-source perception methods perform better in identifying heterogeneous samples and rare cell types.

### Long-tail distribution and optimization strategies for rare cell type recognition

In single-cell type annotation, the long-tail problem is a significant challenge, referring to the relatively limited sample size of rare cell types within the dataset, which reduces model accuracy in identifying these types. This data imbalance not only affects model generalizability but can also result in the loss of important biological information. To address this challenge, scNAME [128] uses a weighted soft K-means clustering algorithm [108] that groups cells toward the most similar centers, while neighborhood contrastive learning [150] minimizes the distance between homologous cells and maximizes the distance between unrelated ones, enabling distinctive representation of rare cells. Meanwhile, scBERT [17], a large-scale pretrained model, employs a bidirectional performer encoder architecture [151, 152] to capture contextual information in cell expression data, deeply learning cell representations so that the model’s attention mechanism focuses on rare cell type distributions, improving the recognition of these cell types.

Although deep learning has made remarkable progress in single-cell annotation, as exemplified by scBERT’s enhancement in recognizing rare cell types, current methods remain constrained by high resource demands and limited efficiency. Firstly, supervised classification models like scBERT depend heavily on large amounts of labeled data, which is particularly challenging for rare cell types where samples are already sparse, limiting model performance on long-tail distributions. Secondly, these models often require substantial computational resources and long pretraining and fine-tuning times, significantly increasing training costs and limiting their applicability in resource-constrained settings. Current deep learning methods show insufficient flexibility and adaptability to effectively meet the demands of rare cell type identification in settings with limited data and dynamic environments.

To improve the recognition accuracy of rare cell types, we propose targeted solutions from three perspectives: data volume, feature representation, and learning difficulty. First, meta-learning [153] and few-shot learning [154] effectively address data scarcity. Meta-learning enables the model to quickly adapt to new tasks, allowing it to identify rare cell types with minimal labeled data, while few-shot learning optimizes model structure to maintain efficient learning even with limited data. Second, for feature representation, attention-based feature selection automatically filters marker genes specific to each cell type, constructing an optimized gene set that more accurately captures key characteristics of rare cell types, alleviating the long-tail distribution problem. Lastly, curriculum learning introduces complex tasks in stages, helping the model progressively grasp features of rare cell types, enhancing learning stability and accuracy. Combined, these strategies significantly enhance model performance in scarce data scenarios, advancing single-cell annotation techniques.

### Exploring the synergy between dynamic clustering and annotation

In the single-cell type annotation task, balancing clustering and annotation has become a critical issue. In the broader context of cell type annotation, once the model identifies known cell types, unseen cell types are marked as “unassigned,” requiring further clustering to identify potential clusters [107]. However, as samples are progressively excluded during the annotation process, the distribution of the remaining data dynamically changes, which affects the stability of clustering, especially when determining the optimal number of clusters. Most existing methods rely on static clustering setups and lack mechanisms for adjusting parameters to account for dynamic changes, making it difficult to optimize clustering number while excluding annotated samples, which ultimately leads to instability in both clustering structure and annotation outcomes. Therefore, the synergy between clustering and annotation is particularly important.

Recent research advancements indicate that clustering methods based on contrastive learning can effectively address this challenge. For instance, scRobust [135], leveraging a self-supervised contrastive learning framework, demonstrates exceptional robustness and adaptability to novel cell types under dynamic data distributions. Experimental results show that in the Zheng 68K dataset, scRobust achieves an identification accuracy of 0.28 for the rare CD4+ T Helper 2 cells, significantly outperforming methods such as Concerto [155], CIForm [106], and TOSICA [13], all of which have accuracy rates below 0.10. Moreover, in the Muraro dataset, scRobust attains a perfect accuracy of 1.0 in identifying epsilon cells, whereas other methods fail to detect this cell type (accuracy = 0). These findings validate the effectiveness of contrastive learning in capturing latent relationships among similar cells, thereby enhancing clustering algorithms’ adaptability to data sparsity and dynamic changes, ultimately providing robust technical support for the efficient identification of unannotated cells.

To address this issue, we propose several strategies to optimize the balance between clustering and annotation. First, adaptive clustering algorithms can dynamically adjust the number and structure of clusters, allowing real-time responses to changes in sample distribution and improving the resolution of previously unseen cell types. Second, automatic clustering optimization based on latent features can be employed, utilizing deep learning to extract cellular features and perform clustering in latent space, ensuring the stability of clustering performance even when data is progressively removed. Finally, contrastive learning-based clustering methods serve as another effective strategy, leveraging advanced models such as scRobust to align global gene information with local features, enabling the capture of multidimensional biological characteristics (e.g. cell subtype-specific pathways and sample-specific markers) in sparse data environments. These strategies not only provide novel technical pathways for dynamic optimization but also establish a foundation for improving annotation accuracy and clustering stability, ultimately achieving a synergistic integration of clustering and annotation.

### Balancing knowledge retention and adaptation in continual learning with the surge in single-cell data

In the context of the rapid accumulation of single-cell sequencing data, continual learning has become a key strategy to enhance the generalization ability and adaptability of single-cell annotation models [88, 89]. With the continuous increase in new sequencing data and cell types, existing models need to be frequently updated. However, direct re-training is time-consuming and may result in forgetting of prior knowledge. The core of continual learning is enabling models to leverage experience gained from previous tasks to help them learn new tasks, thus allowing knowledge to accumulate over time. By progressively absorbing new data, continual learning helps models retain existing knowledge while adapting to new information, thus expanding their recognition capabilities. This approach is particularly suited for handling the rapidly growing single-cell multi-omics data.

In this context, the scEvolve [113] method was proposed to address the continual learning challenges in single-cell annotation. Based on incremental learning principles, it employs prototype comparison and rehearsal learning strategies to mitigate knowledge forgetting. When new data is introduced, scEvolve ensures that the model integrates information on new cell types while maintaining its performance on old cell types by replaying known cell type data. This strategy enhances the model’s adaptability and generalization capabilities, improving both the efficiency and accuracy of single-cell annotation.

However, current research on continual learning in single-cell annotation is still in its infancy, and related methods remain underexplored. There is substantial room for improvement to achieve more robust knowledge expansion and cross-dataset transferability. Therefore, it is necessary to combine some incremental learning strategies [114] to establish a more effective balance between old and new knowledge. Incremental learning emphasizes preserving and optimizing old knowledge while absorbing new knowledge to address the “catastrophic forgetting” problem [156]. For example, knowledge distillation [116] strategies can effectively transfer old knowledge to student models, ensuring they retain the ability to recognize old cell types when absorbing new information, thereby reducing the risk of forgetting. Additionally, dynamic network expansion methods allow models to adjust their network structure when recognizing new cell types, minimizing interference with existing parameters, while regularization methods provide stability constraints to ensure that key weights remain unchanged during updates, helping prevent conflicts between old and new knowledge. Through the combination of these strategies, continual learning in single-cell annotation will have stronger knowledge retention and adaptation capabilities, providing higher accuracy and stability for processing the ever-growing single-cell sequencing data.

### Heterogeneity of unseen cells and their potential decoding from an open-world perspective

From an open-world perspective, one of the core and cutting-edge challenges in single-cell annotation is the effective identification and annotation of unseen cell types. Unseen cell types typically refer to novel cell populations that are absent in the labeled reference dataset but present in the query dataset to be annotated. Notably, while traditional marker gene-based methods are constrained by their dependence on prior knowledge when handling novel cell types, they exhibit distinct advantages in wet-lab validation and cross-platform dataset stability. In particular, under scenarios with significant batch effects, these methods often demonstrate superior interpretability and reliability by leveraging explicit biomarker-based matching. In many biological research contexts, especially within the tumor microenvironment, such novel cell types may contain critical information impacting disease progression or therapeutic responses. Failure to accurately identify these cell types may lead to an incomplete understanding of cellular heterogeneity, potentially overlooking cell populations essential to disease progression and their defining characteristics. Current data-driven methods, such as mtANN [16], scLearn [90], and scBERT [17], employ classification thresholds to label samples below the threshold as “unassigned.” While this dynamic discrimination mechanism expands the scope of recognition, its biological interpretability still requires improvement compared to marker gene-based approaches, which often necessitate manual validation. Particularly when faced with platform-specific variations or technical noise, these two methodological paradigms tend to exhibit complementary strengths: data-driven methods excel at capturing complex expression patterns, whereas marker gene-based approaches, which provide verifiable biological anchors, enhance annotation reliability.

Against this backdrop, hybrid strategies that integrate different technical approaches have emerged as a key area of exploration in open-world single-cell annotation. For instance, scGAD [107] introduces an anchor-pairing strategy, which seamlessly incorporates prior knowledge from reference datasets while preserving the advantages of data-driven learning. This hybrid approach inherits the stability of marker gene-based methods while retaining the sensitivity of machine learning models in detecting novel patterns. Experimental results indicate that this method effectively links reference and target datasets, utilizing known labels to aggregate potential novel cell types. However, purely data-driven methods still face inherent challenges in biological interpretability, particularly in extracting specific gene expression signatures. Existing models often fail to achieve the level of precision required for wet-lab validation, underscoring the necessity of incorporating marker gene validation at critical points in the annotation process.

Future research should focus on developing an integrated framework that combines the strengths of both approaches. One promising direction is to collaboratively validate key genes identified via attention mechanisms against authoritative marker gene databases (e.g. PanglaoDB) [24], establishing a bidirectional closed-loop mechanism of “data-driven discovery: marker gene validation.” This strategy would enhance the interpretability of novel cell cluster features while improving model robustness against batch effects [157]. Furthermore, in exploring adaptive learning algorithms with limited labeled data, a hierarchical validation system inspired by marker gene-based methods could be employed: at the initial screening stage, the model leverages the sensitivity of data-driven approaches, while at the final annotation stage, it incorporates the conservativeness of marker gene-based verification. This layered strategy could significantly enhance the clinical applicability of annotation models. These integrative innovations not only help overcome the technical bottlenecks of individual methods but also pave the way for constructing clinically interpretable intelligent annotation systems, ultimately accelerating the translational application of single-cell analysis technologies in precision medicine.

## Conclusion

This review offers a thorough and comprehensive overview of recent advancements in cell type annotation using scRNA-seq technology, emphasizing the transformative new perspectives it brings to understanding cellular heterogeneity. We systematically analyze and categorize various annotation methods, including those based on specific gene expression, correlation-based reference models, data-driven reference models, and large-scale pretrained models, to evaluate the strengths, weaknesses, and applicability of each approach. To address key challenges such as data sparsity, long-tail distributions, and cellular heterogeneity, we explore the potential of integrating multi-omics data and dynamic clustering algorithms to enhance annotation accuracy and robustness. Moreover, future research should focus on continual learning strategies to improve model adaptability in open-world environments, where the identification of emerging cell types is crucial. Such efforts, supported by robust evaluation frameworks and enabled by interdisciplinary collaboration, will provide a solid foundation for advancing single-cell annotation, thereby shedding light on the pivotal role of cellular complexity in biomedical research.

**Key Points**

- Conduct a comprehensive analysis of various single-cell transcriptome cell type annotation methods, categorizing and elaborating on their characteristics to provide insights for the development of new methods and inspire innovation through cross-method integration.
- Thoroughly examine the experimental evaluation process, covering data processing, preprocessing, evaluation metrics, and performance assessment, optimizing the evaluation framework to assist researchers in improving experimental design and method selection.
- Precisely analyze the challenges in single-cell annotation, focusing on long-tail rare cell identification and the classification of unseen cells in open-world scenarios, while proposing targeted strategies. Highlight the importance of interdisciplinary collaboration, advocate for multi-omics data integration, and encourage the use of dynamic clustering algorithms to enhance continuous learning and foster comprehensive development.
